# Identification of prognostic factors and surgical indications for metastatic gastric cancer

**DOI:** 10.1186/1471-2407-14-409

**Published:** 2014-06-06

**Authors:** Yasuhiko Mohri, Koji Tanaka, Masaki Ohi, Susumu Saigusa, Hiromi Yasuda, Yuji Toiyama, Toshimitu Araki, Yasuhiro Inoue, Masato Kusunoki

**Affiliations:** 1 Department of Gastrointestinal and Pediatric Surgery, Mie University Graduate School of Medicine, Tsu, Japan; 2 Department of Innovative Surgery, Mie University Graduate School of Medicine, Tsu, Japan

## Abstract

**Background:**

The treatment of metastatic gastric cancer is not uniform, and the prognostic factors and indications for surgery are currently unclear. This retrospective study aimed to identify the prognostic factors and clinical indications for surgery in patients with metastatic gastric cancer.

**Methods:**

A total of 123 consecutive patients with gastric cancer and synchronous distant metastasis treated between January 1999 and December 2011 were reviewed. Patient, tumor, laboratory, surgical, and chemotherapy factors were analyzed, with overall survival as the endpoint. Univariate analyses were performed using the log-rank test, multivariate analyses were performed using the Cox proportional hazards model, and Kaplan-Meier curves were used to estimate survival. Significance was set at p < 0.05.

**Results:**

The median overall survival time was 13.1 months. Ninety-eight patients received chemotherapy. Twenty-eight patients underwent gastrectomy with metastasectomy and 55 underwent gastrectomy without metastasectomy. The median overall survival time for patients who underwent gastrectomy with metastasectomy, gastrectomy without metastasectomy, and no surgical intervention was 21.9 months, 12.5 months, and 7.2 months, respectively (p < 0.001). Multivariate analysis identified gastrectomy with or without metastasectomy, performance status (PS) ≥3, neutrophil-to-lymphocyte ratio (NLR) >3.1, and carbohydrate antigen 19–9 (CA19-9) level >37 U/mL as predictors of poor survival. NLR and CA19-9 level were also independent prognostic factors in the group of patients who underwent surgery.

**Conclusions:**

High pretreatment NLR, CA19-9 level, and PS are predictors of poor prognosis in patients with metastatic gastric cancer. In selected patients, gastrectomy can be performed safely, and may be associated with longer survival.

## Background

Gastric cancer is a major health problem. In 2011, 989,600 new cases and more than 738,000 deaths due to gastric cancer were predicted worldwide [1]. Metastatic gastric cancer has a poor prognosis, and the management of this disease is not uniform. In early clinical trials, systemic chemotherapy was associated with longer survival and improved quality of life compared with supportive care alone [2,3]. Currently, the only standard management to prolong survival in patients with metastatic gastric cancer is palliative chemotherapy with best supportive care [4].

The survival benefit of surgical resection (gastrectomy with or without metastasectomy) for metastatic gastric cancer remains unclear. Some studies found that resection may be beneficial in terms of survival, symptomatic relief, and quality of life [5-7], whereas other studies reported poor outcomes after resection [8,9]. No randomized trials comparing resection with observation or other management have been reported. Although there is increasing evidence that chemotherapy for metastatic gastric cancer prolongs survival, the prognosis of metastatic gastric cancer patients who receive only chemotherapy remains poor, with a median overall survival time of about 1 year [10,11].

The aims of this study were to determine the natural clinical course in patients who have metastatic disease at the time of diagnosis with gastric cancer, and to determine the important factors associated with overall survival in terms of the primary tumor and the metastatic disease. Patients who underwent gastrectomy with or without metastasectomy were analyzed separately to identify the factors associated with prolonged survival in this group.

## Methods

From the prospectively collected database at Mie University Hospital, 123 consecutive patients who were diagnosed with metastatic gastric cancer between January 1, 1999 and December 31, 2011 were identified. All patients presented with synchronous primary and metastatic disease prior to treatment. Patient details were recorded at presentation, during all treatments, and at follow-up visits until death or November 2013. Patients who first had metastatic disease diagnosed during laparotomy were excluded from this study.

The Medical Ethics Committee of Mie University Graduate School of Medicine approved this retrospective study. The study was conducted in accordance with the guidelines of the 1975 Declaration of Helsinki. The need for informed patient consent was waived because of the retrospective nature of the study.

The patient characteristics recorded included age, sex, and Eastern Cooperative Oncology Group performance status (PS). Primary tumor data collected included the location of the primary tumor (upper, middle, or lower stomach), degree of differentiation (well, moderate, or poorly differentiated), adjacent organ invasion (present or absent), and bulky perigastric or celiac lymph nodes (present or absent). Laboratory data collected included the neutrophil-to-lymphocyte ratio (NLR; defined as elevated if above the median value of 3.1), hemoglobin (Hb) level (defined as decreased if < 12 g/dL), albumin (Alb) level (defined as decreased if < 3.5 g/dL), C-reactive protein (CRP) level (defined as elevated if >0.2 mg/dL), carcinoembryonic antigen (CEA) level (defined as elevated if >6 ng/mL), and carbohydrate antigen 19–9 (CA19-9) level (defined as elevated if >37 U/mL). Metastatic tumor factors recorded included the number of organs with metastatic disease and the presence or absence of metastasis to the liver, peritoneum, distant lymph nodes, and other organs. NLR was calculated as the neutrophil count divided by the lymphocyte count. Contrast-enhanced computed tomography (CT) was performed to evaluate invasion of the primary tumor into adjacent organs, bulky lymph nodes, and the presence or absence of distant metastasis. Lymph nodes were defined as bulky if an individual node measured ≥3 cm in diameter.

Gastrectomy with or without metastasectomy was considered in patients with adequate organ function and PS ≤ 2. Patients with extensive tumor burden such as extensive peritoneal metastases were not considered suitable for gastrectomy. Patients with severe symptoms such as obstruction, perforation, or bleeding resulting directly from the gastric tumor were considered for gastrectomy without metastasectomy. When baseline CT findings suggested that complete resection was technically feasible, surgery was selected as the initial therapy, and open laparotomy was performed with the aim of achieving complete gross resection of the primary and metastatic tumor. If surgical exploration showed that complete resection was not feasible, the primary tumor was resected and chemotherapy was administered. The extent of surgery was categorized as subtotal gastrectomy, total gastrectomy, extended gastrectomy, or non-resection. The non-resection group included patients who underwent gastric bypass surgery, placement of a feeding jejunostomy tube, and open biopsy. In patients with liver metastasis, complete gross resection was defined as complete removal of hepatic metastases by surgery or ablation. In patients with peritoneal seeding classified as P1 (metastases to the adjacent peritoneum, such as the lesser or greater omentum, but not to the distant peritoneum) or P2 (a few or several scattered metastases to the distant peritoneum) according to the Japanese classification of gastric carcinoma (first English edition), gross resection was defined as complete resection of all peritoneal nodules [12]. In patients with intra-abdominal distant lymph node metastasis, complete gross resection was defined as lymphadenectomy with tumor-free surgical margins. Tumor resection without macroscopic residual cancer at the time of surgery was classified as gastrectomy with metastasectomy, and tumor resection with macroscopic residual cancer was classified as gastrectomy without metastasectomy.

CT for the assessment of treatment response was performed 1 month after the start of chemotherapy and then every 3 months. Patients were reassessed for the feasibility of complete surgical resection at each evaluation. Patient survival was determined by follow-up contact by telephone or mail, or by review of the outpatient records. Patients were followed until death or November 30, 2013. The median follow-up period was 9.3 months.

### Statistical analysis

Data are presented as number (percentage). The clinicopathological factors of the whole group (n = 123) were compared with those of the resection group (n = 83) who underwent gastrectomy with or without metastasectomy. This method was chosen to enable evaluation of prognostic factors with as complete a denominator as possible, and to compare the results with patients who eventually underwent gastrectomy with or without metastasectomy. Patient, tumor, laboratory, and treatment factors were compared between the resection and non-resection groups using the *χ*^2^ test. The end of the follow-up period was November 30, 2013, and the median follow-up period in the resection group was 12.5 months. The beginning of the follow-up period was defined as the date of diagnosis of metastatic gastric cancer. Overall survival was recorded as the time from diagnosis to death regardless of cause, or to the time of the last follow-up (with or without disease). Variables were compared between groups by univariate analyses using the log-rank test, and prognostic factors associated with survival were identified by multivariate analysis using the Cox proportional hazards model with stepwise regression. All analyses were performed using the SPSS computer software package (Statistical Product and Service Solutions 20; SPSS Inc., Chicago, IL, USA). Survival curves were constructed using the Kaplan–Meier method.

## Results

The median survival time of patients with metastatic gastric cancer was 13.1 months. Table 1 shows the frequency distributions of various clinicopathological factors in the whole group (n = 123), the resection group (gastrectomy with or without metastasectomy, n = 83), and the non-resection group (n = 40), including patient, primary tumor, metastatic tumor, laboratory, surgery, and chemotherapy factors.

**Table 1:** Frequency distributions of clinicopathological variables

**Variable**	**Whole group (n = 123)**	**Resection group (n = 83)**	**Non-resection group (n = 40)**	**p value**
Patient data				
Age (years)				
≤ 65	57 (46)	37 (45)	20 (50)	0.670
>65	66 (54)	46 (55)	20 (50)	
Sex				
Female	38 (31)	29 (35)	9 (22)	0.212
Male	85 (69)	54 (65)	31 (78)	
PS				
0	46 (37)	40 (48)	6 (15)	< 0.001
1	47 (38)	34 (41)	13 (33)	
2	20 (16)	9 (11)	12 (30)	
3	10 (9)	0	9 (22)	
Body mass index (kg/m^2^)				
≤ 21	62 (50)	41 (49)	19 (47)	0.848
>21	61 (50)	42 (51)	21 (33)	
Primary tumor data				
Location in stomach				
Lower	31 (25)	24 (29)	7 (18)	0.058
Middle	33 (27)	26 (31)	7 (18)	
Upper	37 (30)	22 (27)	15 (37)	
Whole	22 (18)	11 (13)	11 (27)	
Histological differentiation				
Differentiated	45 (37)	33 (40)	12 (30)	0.324
Undifferentiated	78 (63)	50 (60)	28 (70)	
Adjacent organ invasion				
Present	32 (26)	11 (13)	21 (52)	< 0.001
Absent	91 (74)	72 (87)	19 (48)	
Bulky lymph nodes				
Present	75 (61)	46 (55)	21 (52)	0.079
Absent	48 (39)	37 (45)	19 (48)	
Laboratory data				
CEA (ng/mL)				
≤ 6	76 (62)	49 (59)	27 (68)	0.431
>6	47 (38)	34 (41)	13 (32)	
CA19-9 (U/mL)				
≤ 37	75 (61)	55 (66)	20 (50)	0.114
>37	48 (39)	28 (34)	20 (50)	
NLR				
≤ 3.1	64 (52)	46 (55)	18 (45)	0.337
>3.1	59 (48)	37 (45)	22 (55)	
Hb (g/dL)				
≤ 12	64 (52)	44 (53)	20 (50)	0.114
>12	59 (48)	39 (47)	20 (50)	
CRP (mg/dL)				
≤ 0.2	58 (47)	44 (53)	14 (35)	0.083
>0.2	65 (53)	39 (47)	26 (65)	
Alb (g/dL)				
≤ 3.5	50 (41)	29 (35)	21 (52)	0.079
>3.5	73 (59)	54 (65)	19 (48)	
Metastatic tumor data				
Number of organs involved				
1	74 (60)	53 (64)	21 (52)	0.244
≥2	49 (40)	30 (36)	19 (48)	
Peritoneal metastasis				
Yes	66 (54)	42 (51)	24 (60)	0.343
No	57 (46)	41 (49)	16 (40)	
Distant nodal metastasis				
Yes	55 (45)	30 (36)	25 (62)	0.007
No	68 (55)	53 (64)	15 (38)	
Hepatic metastasis				
Yes	40 (33)	31 (37)	9 (23)	0.107
No	83 (67)	52 (63)	31 (77)	
Surgical data				
Metastasectomy				
Yes	28 (23)	28 (34)	–	
No	95 (77)	55 (66)	–	
Site of metastasectomy			–	
Peritoneum		16	–	
Lymph node		2	–	
Liver		10	-–	
Chemotherapy				
Yes	98 (80)	64 (77)	34 (85)	0.349
No	25 (20)	19 (23)	6 (15)	
Chemotherapy before surgery				
Yes		23 (28)	–	
No		60 (72)	–	
Chemotherapy after surgery				
Yes		64 (77)	–	
No		19 (23)	–	

### Whole group

The median age of patients was 66 years (range 18–94 years) and approximately two-thirds of the patients were male. Ninety patients (73%) died during the follow-up period, with the majority dying of disease-related causes. The most common site of metastasis was the peritoneum (54%), followed by distant lymph nodes (45%) and the liver (33%). There was metastasis to two or more organs in 40% of patients (Table 1). Among patients who did not undergo gastrectomy with or without metastasectomy, 6 received best supportive care only, and 34 received chemotherapy with or without gastric bypass surgery and placement of a feeding jejunostomy tube (see Additional file 1).

Comparisons between the non-resection and resection groups are shown in Table 1. The non-resection group had significantly higher PS, higher frequency of adjacent organ invasion, and higher frequency of distal lymph node metastasis than the resection group.

**Figure 1:**
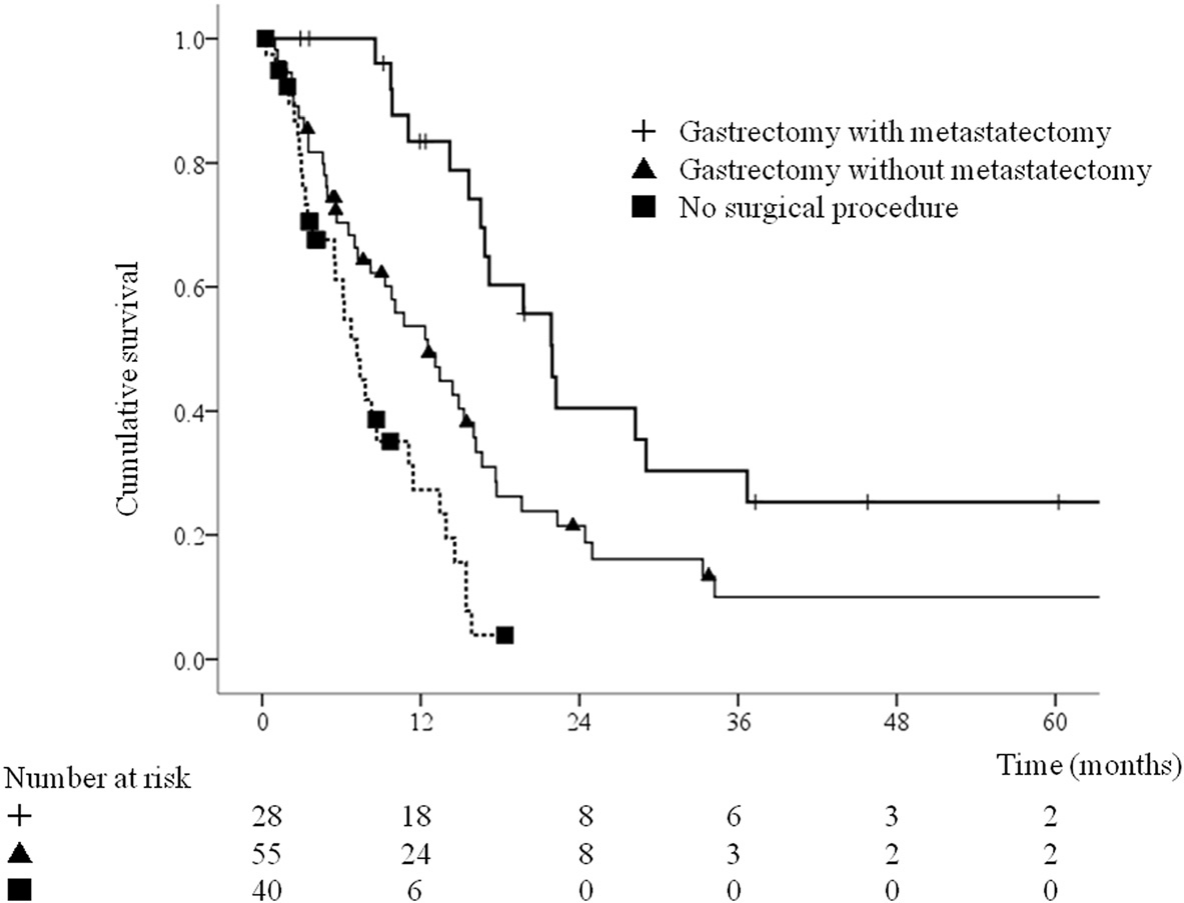
**Overall survival according to surgical procedure (n = 123).** Gastrectomy with metastasectomy, n = 28; gastrectomy without metastasectomy, n = 55; no definitive surgery, n = 40 (p < 0.001).

**Figure 2:**
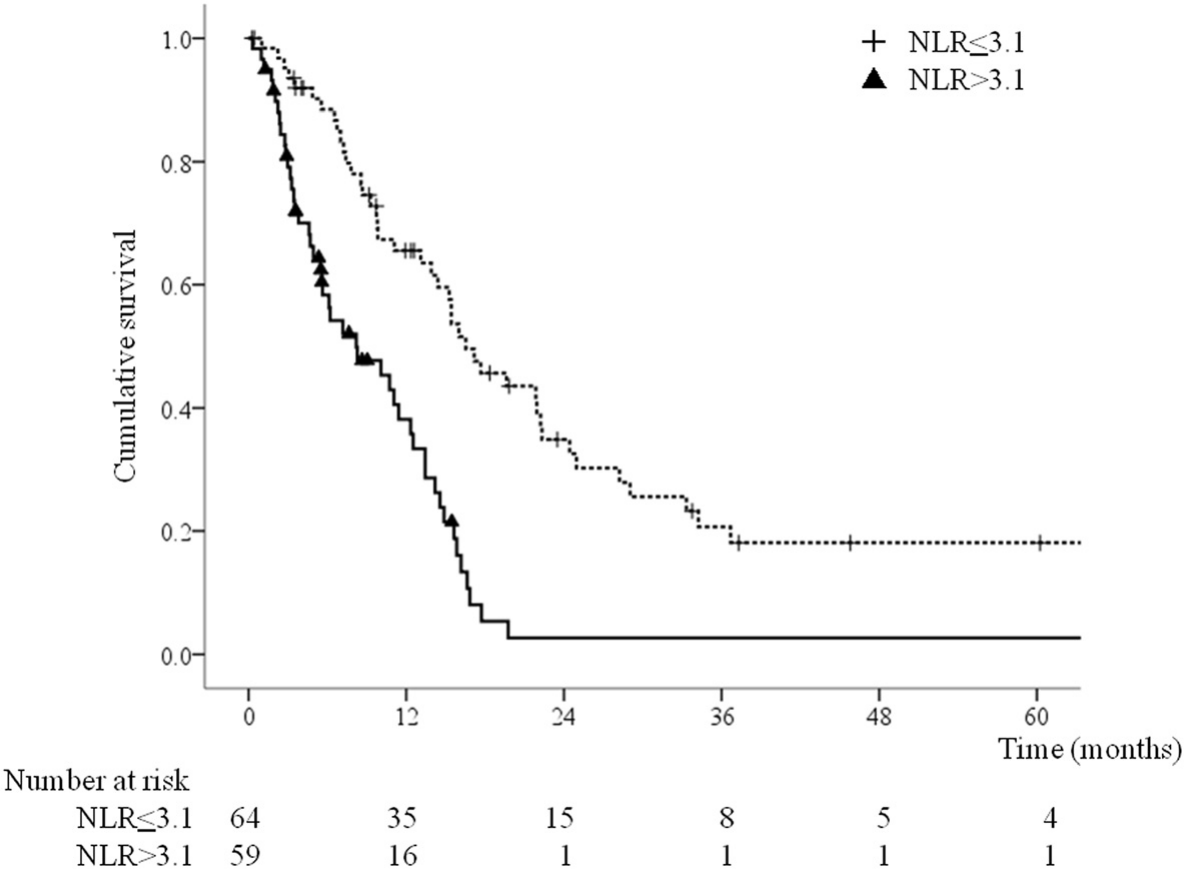
**Overall survival according to neutrophil-to-lymphocyte ratio (NLR) (n = 123).** The NLR was at ≤ 3.1 in 64 patients and >3.1 59 patients (p < 0.001).

**Figure 3:**
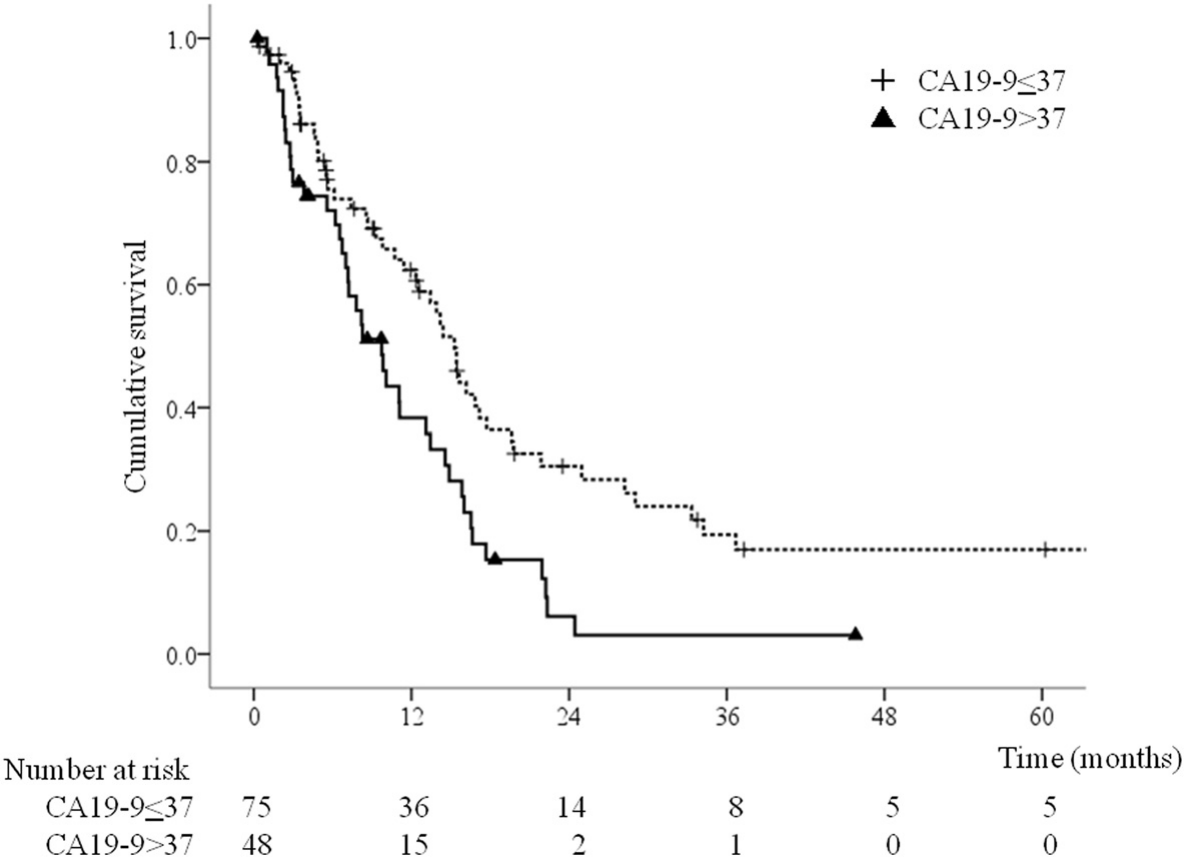
**Overall survival according to CA19-9 level (n = 123).** The CA19-9 level was ≤ 37 U/mL in 75 patients and >37 U/mL in 48 patients (p = 0.003).

Univariate analyses showed that poor survival was significantly associated with PS 3, NLR >3.1, CRP level >0.2 mg/dL, Alb level < 3.5 g/dL, CA19-9 level >37 U/mL, adjacent organ invasion, presence of bulky lymph nodes, metastasis to multiple organs, absence of gastrectomy with or without metastasectomy, and absence of chemotherapy (Table 2). The CEA level tended to be associated with survival, but this association was not significant. Multivariate analysis using the Cox proportional hazards model including the factors associated with survival on univariate analyses (p < 0.05) identified PS ≤ 2, NLR ≤ 3.1, and CA19-9 level ≤ 37 U/mL as significant predictors of longer survival (Table 3). The multivariate model showed longer survival in the resection group compared with the non-resection group [hazard ratio (HR) = 0.55, 95% confidence interval (CI) 0.32–0.95, p = 0.0033) (Table 3).Figure 1 shows that the group who underwent gastrectomy with metastasectomy had the longest overall survival, followed by the group who underwent gastrectomy without metastasectomy, and the group who did not undergo gastrectomy (p < 0.001). The 3-year actuarial survival rate for gastrectomy with metastasectomy, gastrectomy without metastasectomy, and no gastrectomy was 25.3%, 10.1%, and 0%, respectively. Only patients who underwent gastrectomy with or without metastasectomy survived for longer than 5 years. Figure 2 shows the unfavorable effect of NLR >3.1 (p < 0.001) and Figure 3 shows that CA19-9 level >37 U/mL was associated with poorer survival (p = 0.003).

**Table 2:** Univariate analyses for overall survival in metastatic gastric cancer patients (n = 123)

**Variable**	**Median survival (months)**	**p value**
Age (years)		0.362
>65	13.4	
< 65	13.1	
Sex		0.583
Female	11.1	
Male	14.2	
PS		< 0.001
0, 1, 2	14.2	
3	2.4	
Body mass index (kg/m^2^)		0.242
< 21	11.1	
>21	14.9	
Hb (g/dL)		0.428
< 12	13.4	
>12	13.1	
NLR		< 0.001
< 3.1	16.5	
>3.1	8.2	
CRP (mg/dL)		0.005
< 0.2	15.4	
>0.2	9.8	
Alb (g/dL)		< 0.001
< 3.5	6.7	
>3.5	15.6	
CEA (ng/mL)		0.052
< 6	14.2	
>6	9.7	
CA19-9 (U/mL)		0.003
< 37	15.3	
>37	9.7	
Tumor location in stomach		0.267
Upper	13.4	
Middle	12.3	
Lower	14.2	
Whole	7.4	
Histological differentiation		0.829
Differentiated	14.6	
Undifferentiated	11.4	
Adjacent organ invasion		0.009
Yes	7.8	
No	14.6	
Bulky lymph nodes		0.011
Yes	9.3	
No	12.5	
Metastasis to organs		0.044
1 organ	15.4	
≥2 organs	10.1	
Peritoneal metastasis		0.174
Present	11.1	
Absent	16.2	
Hepatic metastasis		0.556
Present	15.3	
Absent	11.4	
Distant lymph node metastasis		0.117
Present	10.1	
Absent	14.6	
Gastrectomy		< 0.0001
Present	15.6	
Absent	7.2	
Chemotherapy		0.007
Yes	14.4	
No	4.7	

**Table 3:** Multivariate analysis for overall survival in metastatic gastric cancer patients (n = 123)

**Variable**	**HR**	**95% CI**	**p value**
PS 3	8.69	3.45–21.87	< 0.001
NLR >3.1	2.30	1.44– 3.67	< 0.001
CA19-9 > 37 U/mL	1.77	1.14–2.76	0.012
Bulky lymph nodes	1.53	0.98–2.39	0.063
Gastrectomy with or without metastasectomy	0.55	0.32–0.95	0.033

### Surgery group

Eighty-three patients underwent gastrectomy with or without metastasectomy, of which 47 (57%) underwent total gastrectomy and 24 (29%) underwent partial gastrectomy. Twelve patients (14%) underwent *en bloc* resection of the tumor with an adjacent organ, most commonly the spleen or distal pancreas. Thirty-six patients (43%) underwent D2 or more extensive lymphadenectomy. Twenty-eight patients who underwent metastasectomy, including 2 (7%) who underwent resection of para-aortic lymph nodes metastasis, 10 (36%) who underwent hepatectomy and/or ablation of hepatic metastasis, and 16 (57%) who underwent peritonectomy for peritoneal metastasis (Table 1). Postoperative complications including wound infection, intra-abdominal abscess, leakage, and small bowel obstruction were not severe in most cases, and there were no surgery-related perioperative deaths.

Twenty-three of the 83 patients (28%) received systemic chemotherapy prior to surgery, including 15 who received 5-fluorouracil and cisplatin, 6 who received taxane and 5-fluorouracil, and 2 who received irinotecan and cisplatin. In these 23 patients, the median time from the diagnosis of metastatic disease to surgery was 1.9 months (range 1–13.6 months). Five of these 23 patients underwent planned gastrectomy without metastasectomy because of gastric obstruction, bleeding, or perforation. In these five patients, the median time from diagnosis to surgery was 0.6 months. In the remaining 18 patients, complete resection was planned. Thirteen of these 18 patients underwent successfully gastrectomy with metastasectomy (complete resection), and the remaining 5 underwent gastrectomy without metastasectomy because surgical exploration revealed an unexpectedly large metastatic tumor burden. In these 18 patients, the median time from diagnosis to surgery was 3.8 months.

Sixty patients underwent initial surgery for the primary and metastatic tumors. Of these, 29 underwent planned gastrectomy without metastasectomy for symptom palliation (obstruction or bleeding). Complete resection was planned in the remaining 31 patients, who did not have obvious symptoms caused by the gastric cancer. Fifteen of these 31 patients (48%) underwent gastrectomy with metastasectomy, and 16 underwent gastrectomy without metastasectomy because surgical exploration revealed an unexpectedly large tumor burden.

All patients who underwent gastrectomy with metastasectomy received postoperative chemotherapy. Nineteen of the 55 patients who underwent gastrectomy without metastasectomy did not receive postoperative chemotherapy because of the patient’s decision or decreased organ function.

The median survival time in patients who underwent gastrectomy with and without metastasectomy was 21.7 and 12.7 months, respectively (Figure 1). Patients who underwent gastrectomy with metastasectomy had significantly longer survival than patients who underwent gastrectomy without metastasectomy. Sixty patients (72%) died during the follow-up period, all from disease-related causes. Ten of the patients (36%) who underwent gastrectomy with metastasectomy had no evidence of tumor recurrence at the time of the last follow-up (median follow-up period 29.4 months, range 12.2–60.2 months). Univariate analyses showed that poor survival was significantly associated with NLR >3.1, CRP level >0.2 mg/dL, Alb level < 3.5 g/dL, CEA level >6 ng/mL, CA19-9 level >37 U/mL, absence of metastasectomy, and absence of chemotherapy (Table 4). The number of organs with metastatic disease tended to be associated with survival, but this association was not significant. Multivariate analysis using the Cox proportional hazards model including the factors associated with survival on univariate analyses (p < 0.05) identified NLR >3.1 (HR = 2.11, 95% CI 1.06–4.22, p = 0.034), and CA19-9 level ≤ 37 U/mL (HR = 2.31, 95% CI 1.22–4.36, p = 0.010) as significant predictors of longer survival (Table 5).

**Table 4:** Univariate analyses for overall survival in metastatic gastric cancer patients who underwent surgery (n = 83)

**Variable**	**Median survival (months)**	**p value**
Age (years)		0.269
>65	16.0	
< 65	15.6	
Sex		0.211
Male	16.6	
Female	11.1	
Body mass index (kg/m^2^)		0.647
>21	17.2	
< 21	14.2	
Hb (g/dL)		0.423
>12	17.2	
< 12	14.4	
NLR		< 0.001
>3.1	21.9	
< 3.1	11.1	
CRP (mg/dL)		0.016
>0.2	11.1	
< 0.2	17.2	
Alb (g/dL)		0.001
>3.5	17.7	
< 3.5	9.8	
CEA (ng/mL)		0.022
≤ 6	16.8	
>6	13.4	
CA19-9 (U/mL)		0.001
≤ 37	17.7	
>37	10.1	
Tumor location in stomach		0.426
Upper	16.2	
Middle	15.6	
Lower	16.0	
Whole	13.1	
Adjacent organ invasion		0.364
Yes	13.1	
No	16.2	
Bulky lymph nodes		0.149
Yes	13.4	
No	17.7	
Histological differentiation		0.404
Differentiated	16.0	
Undifferentiated	15.6	
Metastasis to organs		0.078
1 organ	17.7	
≥2 organs	14.2	
Peritoneal metastasis		0.213
Yes	12.5	
No	17.7	
Hepatic metastasis		0.784
Yes	16.5	
No	14.4	
Distant lymph node metastasis		0.973
Yes	14.9	
No	16.5	
Surgical procedure		0.017
Gastrectomy	12.5	
Gastrectomy + metastasectomy	21.9	
Chemotherapy		0.015
Yes	16.6	
No	8.2	

**Table 5:** Multivariate analysis for overall survival in metastatic gastric cancer patients who underwent surgery (n = 83)

**Variable**	**HR**	**95% CI**	**p value**
NLR >3.1	3.16	1.81–5.51	< 0.001
CA19-9 > 37 U/mL	2.65	1.55–4.52	< 0.001

## Discussion

The results of this study demonstrate that gastrectomy with or without metastasectomy prolongs survival in a highly selected group of patients with metastatic disease at the time of presentation with gastric cancer, compared with patients who do not undergo surgical intervention. Many previous studies have evaluated surgical resection for metastatic gastric cancer, but this study evaluated surgical intervention specifically in patients with metastatic disease at the time of presentation, compared with patients at the same institution who either were not referred for surgical resection or were evaluated but were not considered to be suitable for surgical resection. Understanding that there is a selection bias, comparison of the survival curve of the non-surgical group (patients who were not candidates for surgical intervention and patients who may have been surgical candidates but were not offered surgery) with the survival curve of the surgical group suggests that surgical intervention has a favorable effect on survival. In our entire cohort, the factors identified as predictors of longer survival on multivariate analysis were PS ≤ 2, NLR ≤ 3.1, gastrectomy with or without metastasectomy, and CA19-9 level ≤ 37 U/mL. Separate analysis of the surgical group showed that NLR and CA19-9 level were the most important factors associated with survival in this group.

Generally, the reasons for performing gastrectomy with or without metastasectomy in gastric cancer patients with distant metastasis are: (1) primary tumor resection to relieve potentially life-threatening symptoms such as obstruction, perforation, or bleeding; (2) increased responsiveness of the residual tumor to adjuvant treatment after removal of a significant proportion of the tumor load; and (3) potential immunological benefits because of reduction of immunosuppressive cytokines produced by the tumor [13-15]. Gastrectomy is the procedure of choice in selected patients, even though it has never been compared with observation in a randomized trial. Multiple previous studies reported that gastrectomy with or without metastasectomy prolonged survival in patients with metastatic gastric cancer [16,17]. In our study group, the indications for surgical intervention were: (1) adequate organ function and acceptable PS, (2) absence of extensive invasion of the primary tumor into adjacent organs, and (3) absence of extensive metastatic tumor. Our results are in general agreement with those of previously reported studies, suggesting that our indications for surgery are feasible, and that surgical intervention is beneficial for patients with metastatic gastric cancer.

Over the past few decades, several studies have attempted to identify the prognostic factors in patients with metastatic gastric cancer. In general, it is thought that greater residual tumor load and higher PS negatively affect prognosis. However, the associations between prognosis and pretreatment laboratory data have not been fully determined. This study identified pretreatment NLR and CA19-9 level as prognostic factors in patients with metastatic gastric cancer. CEA and CA19-9 levels reflect tumor biology and are commonly used markers for gastric cancer [18]. CA19-9 may play a role in the adhesion of cancer cells to endothelial cells, resulting in hematogenous metastasis [19]. Immunohistochemical examination showed marked expression of CA19-9 in gastric cancer tissue [20]. One study reported that CEA and CA19-9 levels were associated with prognosis in patients with gastric cancer who had undergone curative resection [21]. Another study found that elevated CA19-9 levels in gastric cancer patients were well correlated with various types of metastasis [22]. This study identified a high pretreatment CA19-9 level as an independent prognostic factor. On the other hand, it is increasingly recognized that clinical outcomes in cancer patients are influenced not only by the oncological characteristics of the tumor, but also by host-response factors. It has been suggested that NLR (calculated as neutrophil count divided by lymphocyte count), CRP level, and albumin level reflect host-response factors in various solid tumors including gastric cancer. This study found that an elevated NLR was an independent prognostic factor in patients with metastatic gastric cancer. Interestingly, NLR and CA19-9 level were independent prognostic factors both in the overall group of patients with metastatic gastric cancer and in the group of patients who underwent surgical resection. We therefore suggest that the pretreatment NLR and CA19-9 level can be used to select patients who are suitable for surgery.

Local treatment modalities such as gastrectomy, metastasectomy, ablation therapy, or a combination of these may effectively manage tumor burden. However, many clinicians have concerns about the detrimental effects of surgery in patients with metastatic gastric cancer. Even in large volume centers, gastrectomy for metastatic gastric cancer has been reported to be associated with high rates of morbidity (>50%) and mortality (6–12%) [7,23]. Some recent studies [24,25] reported acceptable postoperative morbidity and mortality rates. In this study, severe postoperative morbidity was uncommon and there were no surgery-related perioperative deaths. The results of some previous studies and of this study therefore indicate that gastrectomy with or without metastasectomy can be safely performed at institutes with appropriate experience.

Previous studies [10,11] reported that systemic chemotherapy improves survival, and chemotherapy has therefore been the mainstay of treatment for metastatic gastric cancer. However, there is ongoing controversy regarding the usefulness of surgical resection for metastatic gastric cancer, the indications for surgery, and the type of surgery that should be performed. A previous study that reported good outcomes after surgical resection, including good survival outcomes, was limited by the selection of patients with less severe disease for surgical resection. The current study therefore made an effort to eliminate selection bias. First, preoperative CT findings were reviewed to determine the preoperative stage of all patients. Second, patients were stratified according to the presence or absence of chemotherapy. Although chemotherapy was found to be significantly associated with prognosis in the whole group on univariate analysis, it was not found to be an independent prognostic factor on multivariate analysis. The prognostic effect of chemotherapy was therefore minimal in this study.

Although the role of metastasectomy is well established for colorectal cancer and sarcoma, there is still controversy regarding the usefulness of surgery targeting metastatic lesions in patients with gastric cancer, who have a reported median survival time of 11.2–31.0 months [24,26]. Some non-randomized comparative analyses suggested that aggressive surgical treatment of patients with metastatic gastric cancer prolongs survival. However, metastatic gastric cancer encompasses a heterogeneous patient population in which both palliative and curative treatment strategies may be used. In the current study, patients who underwent gastrectomy with metastasectomy had a much longer survival time than patients who underwent gastrectomy without metastasectomy. Although only 28 patients underwent gastrectomy with metastasectomy, this included 13 patients who initially had an unresectable tumor burden. The data from this study suggest that gastrectomy with metastasectomy may improve outcomes patients with metastatic gastric cancer selected according to the NLR and CA19-9 level.

In this study, patients who underwent tumor resection had significantly longer survival times than those who did not. However, this result must be interpreted with caution because of the retrospective nature of the study and the differences in patient characteristics between the two groups. Decisions regarding suitability for resection are strongly influenced by invasion of neighboring organs, the number of organs with metastasis, and PS. In this study, patients who underwent gastrectomy with or without metastasectomy had a better PS and were more likely to have no neighboring organ invasion than patients who did not undergo gastrectomy. It has been suggested that this selection bias is the most important contributor to the difference in survival between the two groups. Although the depth of invasion and the number of organs with metastasis were not found to be independent predictors of survival on multivariate analysis, the survival benefit from gastrectomy with or without metastasectomy should be further evaluated by stratified analysis. Recently, prospective randomized trials (the Japan Clinical Oncology Group [JCOG] 0705 and Korea Gastric Cancer Association [KGC] A01 and GYMSA trials) were initiated to evaluate the role of debulking gastrectomy in patients with metastatic gastric cancer [27,28]. These randomized trials are expected to clarify the role of debulking gastrectomy in this patient population.

## Conclusions

The results of this study show that gastrectomy with or without metastasectomy for gastric cancer can be performed safely and is associated with longer survival compared with a nonrandomized control group treated during the same period at the same institution. It is not known whether this is due to differences in PS or disease burden between the two patient groups. A prospective randomized trial could help to determine whether gastrectomy should be considered in selected patients with metastatic gastric cancer. Surgeons should carefully consider surgical intervention in patients with an elevated NLR or CA19-9 level, because these patients have a poor prognosis with or without surgical intervention. Evaluation of novel combinations of resection, local ablation, and chemotherapy should also continue. Gastrectomy with or without metastasectomy, performed safely and in addition to other available treatments, is an important aspect of the multidisciplinary management of patients with metastatic gastric cancer. A larger prospective trial is needed to further evaluate surgery for the treatment of metastatic gastric cancer.

## Abbreviations

Alb: Albumin; CA19-9: Carbohydrate antigen 19–9; CEA: Carcinoembryonic antigen; CRP: C-reactive protein; NLR: neutrophil-to-lymphocyte ratio; PS: Eastern Cooperative Oncology Group performance status.

## Competing interests

The authors declare that they have no competing interests.

## Authors’ contributions

YM, KT and MK conceived and designed the study. YM, KT, MO, SS, HY, YT, TA and YI acquired the data. YM, KT, MO, SS, HY, YT, TA, YI and MK analyzed and interpreted the data. YM, KT, MO, SS, HY, YT, TA, YI and MK drafted the manuscript. YM, KT, MK critically revised the manuscript. All authors read and approved the final manuscript.

## Pre-publication history

The pre-publication history for this paper can be accessed here:

http://www.biomedcentral.com/1471-2407/14/409/prepub

## Supplementary Material

Additional file 1 **Evaluation and treatment flow in 123 metastatic gastric cancer patients.** Twenty-nine patients underwent gastrectomy without metastasectomy for symptom palliation. Thirty-one patients were initially judged to have resectable disease. Twenty-three of the 63 patients who were initially judged to have unresectable disease underwent gastrectomy with or without metastasectomy after chemotherapy. Click here for file
