# Supplementary figures and images for: Identification of prognostic factors and surgical indications for metastatic gastric cancer
Source: BMC Cancer. 2014 Jun 6;14:409. doi: 10.1186/1471-2407-14-409 (PMC4057566; doi:10.1186/1471-2407-14-409)

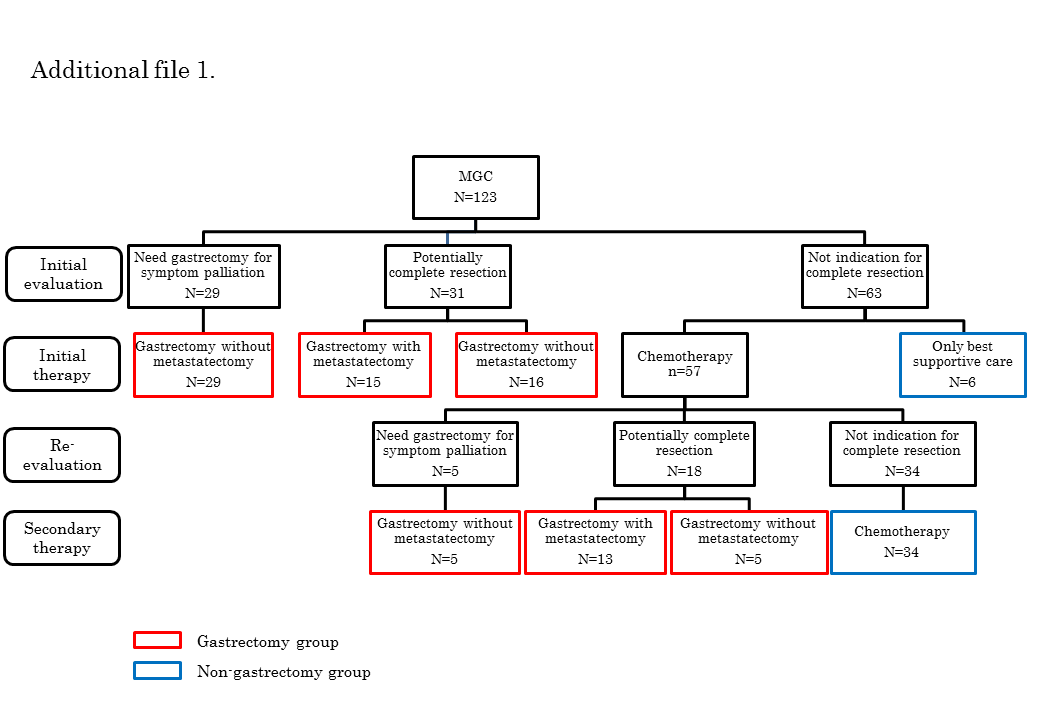

Supplement: Additional file 1 — Evaluation and treatment flow in 123 metastatic gastric cancer patients. Twenty-nine patients underwent gastrectomy without metastasectomy for symptom palliation. Thirty-one patients were initially judged to have resectable disease. Twenty-three of the 63 patients who were initially judged to have unresectable disease underwent gastrectomy with or without metastasectomy after chemotherapy. [file 1471-2407-14-409-S1.tiff]
